# Lymphoepithelial Carcinoma Originated from the Sinonasal Cavity: Case Report and Literature Review

**DOI:** 10.1155/2023/4217102

**Published:** 2023-05-17

**Authors:** Hassan Alhazzani, Saleh Alabood, Ahmed Alhussien, Sahar Alsadah, Abdulrahman Alghulikah, Shuaa Asiri, Ibrahim Alarifi

**Affiliations:** ^1^College of Medicine, King Saud University, Riyadh, Saudi Arabia; ^2^Otolaryngology–Head & Neck Surgery Unit, Surgery Department, Security Forces Hospital Program, Riyadh, Saudi Arabia; ^3^Otolaryngology–Head & Neck Surgery Department, College of Medicine, King Saud University, Riyadh, Saudi Arabia; ^4^Otolaryngology–Head & Neck Surgery Department, Prince Mohammed Bin Abdulaziz Hospital, Riyadh, Saudi Arabia; ^5^Pathology and Laboratory Medicine Department, Security Forces Hospital, Riyadh, Saudi Arabia

## Abstract

**Background:**

Sinonasal lymphoepithelial carcinoma (SNLEC) is a rare neoplasm, representing less than 1% of all types of carcinomas and approximately 3% of head and neck tumors. It can affect the nasopharynx due to the rich lymphoid tissue present in this region. Clinical SNLEC presentation varies, ranging from asymptomatic to nonspecific sinonasal symptoms. We report a case of SNLEC and review the literature for SNLEC presentation, diagnosis, management options, and outcomes. *Case Presentation*. A 38-year-old male, medically free, presented to the emergency department complaining of nasal obstruction, right facial numbness, persistent right-sided headache, intermittent orbital pain, and a history of on/off epistaxis. Imaging showed a destructive mass in the right sphenoid sinus extending to different sinuses and infratemporal fossa. Biopsy confirmed the diagnosis of SNLEC, with immunohistochemistry being positive for Epstein–Barr virus (EBV) and CK8/18. Induction chemotherapy was started with three cycles of cisplatin and gemcitabine, followed by concurrent chemoradiation therapy.

**Conclusion:**

SNLEC is rare, with limited reported cases from around the world. It is mostly seen in adults between their fifth and seventh decades with male predominance. SNLEC is diagnosed using imaging, immunohistochemistry, and EBV testing given its strong association with EBV. Owing to the limited cases, there is no standard approach to treating SNLEC. However, most cases managed with radiation and with and without other modalities showed an excellent response in terms of tumor nonrecurrence.

## 1. Introduction

Sinonasal lymphoepithelial carcinoma (SNLEC) was first independently reported in 1921 by Regaud and Schmincke [1]. It is described by the World Health Organization as “a poorly differentiated squamous cell carcinoma or histologically undifferentiated carcinoma accompanied by a prominent reactive lymphoplasmacytic infiltrate, morphologically similar to nasopharyngeal carcinoma” [2]. SNLEC has been reported to occur in the oral cavity, oropharynx, nasopharynx, larynx, salivary glands, paranasal sinuses, and other organs in the head and neck region [3]. SNLEC represents less than 1% of all types of carcinomas and approximately 3% of head and neck tumors [4]. Its clinical presentation varies, ranging from asymptomatic to nonspecific sinonasal symptoms, which raises a challenge for both the diagnosis and management of SNLEC. We report a case of an adult male with SNLEC of the right nasal cavity, as well as the results of a literature search using Web of Science, PubMed, and Google Scholar. We reviewed the articles to extract information on SNLEC presentation, diagnosis, management options, and outcomes.

## 2. Case Presentation

**Figure 1:**
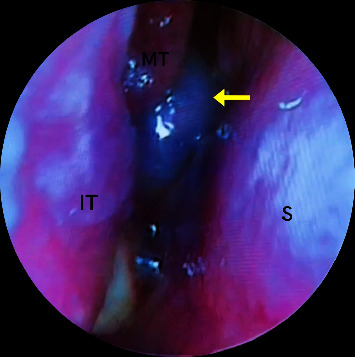
Endonasal scope of the right nasal cavity showed a dark-looking mass (yellow arrow) with purulent discharge under being mixed with blood stain. S: nasal septum; IT: inferior turbinate; MT: middle turbinate.

**Figure 2:**
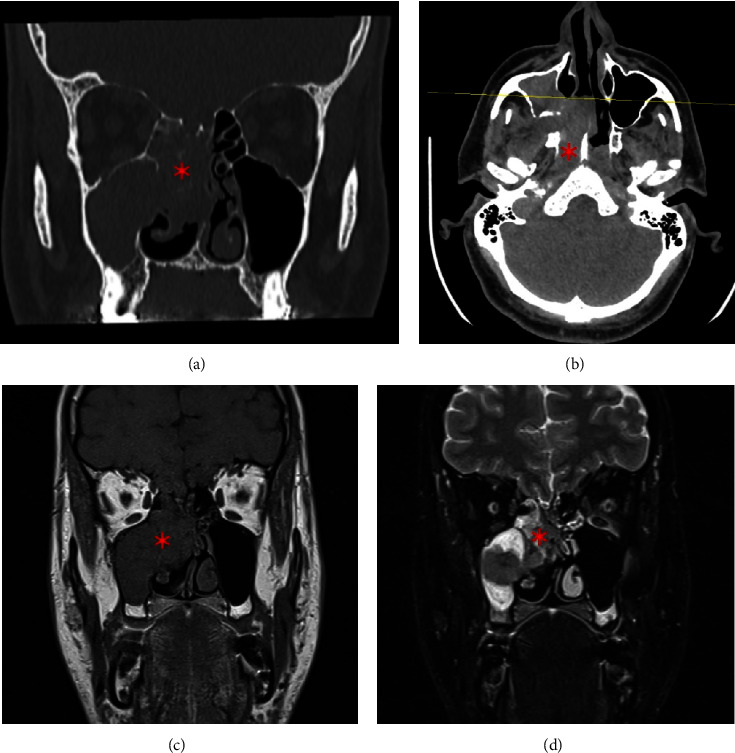
Computed tomography scans (upper left and right) and magnetic resonance imaging T1 (lower left) and T2 (lower right) of the destructive mass (red asterisk) in the right sphenoid sinus extending to different sinuses and the infratemporal fossa.

**Figure 3:**
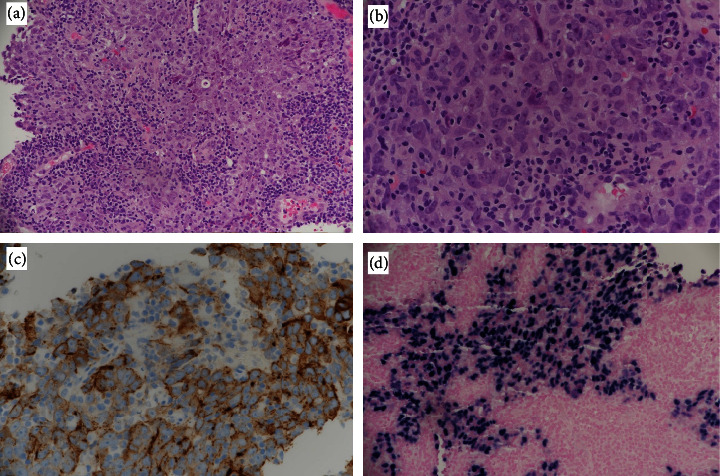
Photomicrographs of the nasopharyngeal biopsy showing (a) infiltrative malignant neoplasm admixed with mixed chronic inflammatory cell infiltrate (H&E stain; 10x); (b) tumor cells with moderate amphophilic cytoplasm, vesicular nuclei, and prominent nucleoli; (c) lymphoplasmacytic cell infiltrate (arrow; H&E stain; 20x), with tumor cells showing cytoplasmic positivity for CK8/18 (IHC; 20x); (d) tumor cells with nuclear positivity for EBER (in situ hybridization for Epstein–Barr virus; 20x).

A 38-year-old male with a history of heavy smoking (20 cigarettes per day for the last 20 years), otherwise healthy, presented to the emergency department in June 2022 complaining of nasal obstruction, right facial numbness, persistent right-sided headache, intermittent orbital pain, and a history of recurrent epistaxis. His surgical history included septoplasty three months prior to his presentation, and his symptoms started shortly after the procedure. Nasal endoscopy revealed a mass filling in the nasal cavity on the right side, and a physical exam was otherwise unremarkable (Figure 1). A computed tomography (CT) scan of the paranasal sinus revealed opacification and expansion of the right sphenoidal sinus by a poorly defined soft tissue mass that extended into the nasal cavity and eroded the inferior sphenoidal bony wall. Magnetic resonance imaging (MRI) of the facial area showed a large enhancing destructive mass extending along the sphenoid, posterior ethmoidal, and right maxillary sinuses, with the extension along the posterior aspect of the nasal cavity and the right infratemporal fossa with an apparent large right retropharyngeal lymph node with significant enhancement in the postintravenous contrast (Figure 2). After that, biopsy confirmed a SNLEC diagnosis. Immunohistochemistry findings were positive for Epstein–Barr virus (EBV) and CK8/18 and negative for CK5/6, P63, chromogranin, synaptophysin, and CD56 (Figure 3). A positron emission tomography (PET) scan was performed which showed a nonspecific moderate activity in the right nasal cavity. Also, there is a moderately increased metabolic activity corresponding to a lymph node in the right neck in the jugular digastric chain at level 3 which is nonspecific and could represent an inflammatory versus neoplastic lesion. For the chest, abdomen, pelvis, and skeleton, there were no focal activities suggestive of distant metastasis.

Tumor grading revealed a T4N2M0 tumor categorized as stage IVa. After the induction chemotherapy with three cycles of cisplatin and gemcitabine, the patient tolerated the therapy sessions with minimal side effects, and repeated imaging showed a reduction of tumor size from 6.7 × 3.4 to 4.7 × 2.6 cm. As result of this good initial response, concurrent chemoradiation therapy started, and the patient is currently under treatment.

## 3. Discussion

SNLEC is still globally rare, with limited clinical studies conducted on this topic. Other names have also been attributed to this tumor, such as undifferentiated carcinoma with lymphoid stroma, lymphoepithelioma, and lymphoepithelial-like carcinoma [5]. SNLEC tends to arise in organs that are rich in lymphoid tissue, including, but not exclusive to, the nasopharynx [6]. Clinical SNLEC presentation is widely variable, ranging from cases that are asymptomatic and found incidentally to cases with nonspecific chief complaints such as headache, nasal congestion, anosmia, and epistaxis. These symptoms may mimic any other sinonasal pathology, making it challenging for otolaryngologists to reach a final diagnosis on the first encounter [6–9].

Upon suspicion of SNLEC, immunohistochemical testing is important to rule out other differential diagnoses such as melanoma, lymphoma, and sinonasal undifferentiated carcinoma (SNUC). Another way to differentiate SNLEC is that it is not immunoreactive to hematolymphoid, melanoma-related, and neuroendocrine/neuroectodermal markers, as well as stains positive for EMA [10]. SNUC is also EBV-negative and has a uniquely aggressive clinical disease course histopathologically characterized by prominent tumor necrosis and apoptosis with low syncytial nuclei quality, which differentiates it from nasopharyngeal squamous cell carcinoma and SNLEC [5]. From the few reported cases, SNLEC is more prevalent in areas where EBV is endemic, such as Southeast Asia. However, cases from the United States and Western Europe tend to present as EBV-negative [11].

To the best of our knowledge, SNLEC has been reported in a countable number of cases worldwide; eight of the 13 cases we reviewed were EBV-positive (Table 1). Nevertheless, Zong et al. published data on 20 cases from Guangzhou, China, collected from 1989 to 1996, all of which were EBV-positive [7]. SNLEC is also mostly seen in adults between their fifth and seventh decades at a male predominance of 3 : 1 [2]. In our literature review of 13 published cases, we found the mean age of presentation to be 52.9 years, with the youngest presentation at 21 years and the oldest at 77 years. The male-to-female ratio was 8 : 5 (Table 1).

Owing to the limited number of reported cases, there is no standard approach to treating SNLEC. Dubey et al. suggested that the tumor is highly radiosensitive and responds well to radiation, making radiation a sufficient means of achieving tumor control [20]; they also indicated that distant metastasis is the main cause of radiation failure. Most reported cases similarly prescribed radiation for tumor management (10 patients), though other treatment modalities were either surgical or chemotherapy and tailored to tumor location, stage, and the presence or absence of metastases (Table 1).

Moreover, SNLEC tends to spread locally; neck and distant metastases are not common, with two out of the 13 cases we reviewed having positive neck lymph nodes [10, 14]. Only one case had distantly metastasized to the spine, which was managed with cisplatin and docetaxel and had excellent tumor response after two cycles [10]. Of the reported cases, six were managed by chemotherapy plus radiotherapy and one was managed by chemotherapy alone with different regimens; all of them showed no signs of tumor recurrence after follow-ups ranging from 13 months to five years. Surgical intervention (accompanied by chemoradiotherapy or radiotherapy) was done in various manners, including Enbloc resection with medial maxillectomy, Danker's operation, and excision, with no tumor recurrence in follow-up.

## 4. Conclusion

SNLEC is a very rare type of cancer with limited reported cases from around the world. It is mostly seen in adults between their fifth and seventh decades with male predominance and diagnosed using imaging and immunohistochemistry. Owing to the limited cases, there is no standard approach to treating SNLEC. However, most cases managed with radiation with and without other modalities have shown an excellent response in terms of tumor nonrecurrence.

## Figures and Tables

**Table 1:** Summary of previously reported cases of sinonasal lymphoepithelial carcinoma (SNLEC).

Authors	Age/gender	Race	Site of lesion	IHC	Stage	Surgery	Radiation	Chemotherapy	Survival/recurrence
Hajiioannou et al. [6]	33/M	NR	Nasal cavity, ethmoid sinus with frontal lobe invasion	+p40, rare focal	T4bN0M0 stage IVB	No	66 Gy	5-fluorouracil and cisplatin	Complete tumor response
				+p63, rare focal + EBV					
				+Cytokeratins					
				MNF116 and EMA					

Rytkönen et al. [12]	30/M	NR	Maxillary sinus, soft palate/uvula with regional LN metastases	+Cytokeratin	T2N0M0 stage II	Yes	70 Gy	Cisplatin	11-month follow-up, no recurrence
				+CD45 (among lymphoplasmacytic infiltrate)					
				+AE1/AE3					
				−EBV					

Tam et al. [13]	61/F	NR	Nasolacrimal duct	+EBV	NR	Enbloc resection with medial maxillectomy	64 Gy	No	33-month follow-up, no recurrence

Takakura et al. [14]	63/M	Japanese	Maxillary sinus	+Pancytokeratin, cytokeratin 14	T3N2bM0 stage IVA	Denker's operation	36 + 34 Gy	5-Fluorouracil and nedaplatin	Disease-free for 5 years following operation
				+EBV					

Mohammed et al. [8]	72/F	Caucasian	Maxillary sinus	+Pancytokeratin marker (MF116) +CK5/6, slight	T1N0M0 stage I	Radical excision of the mass	48 Gy	No	3-years follow-up, no recurrence
				−CK7, CEA, Melan, CK20, EBV					

Jung et al. [15]	64/F	Asian	Maxillary sinus	+Pancytokeratin	T3N0M0 stage III	Enbloc sparing periorbita	6300 cGy	Docetaxel and carboplatin	3-year-follow-up, no recurrence
				−EBV					

Muthayam et al. [16]	45/F	Indian	Maxillary sinus with LN metastases	+Pancytokeratin, diffuse	T3N0M0 stage III	No	70 Gy	No	20-month follow-up, no recurrence
				−Melanin A, CK20, EBV					

Kim et al. [17]	21/M	NR	Nasal cavity	+Cytokeratin, CK5/6	T2N0M0 stage II	Endoscopic surgical excision	IMRT, 6996 cGy	Cisplatin	15-month follow-up, no recurrence
				+EBV					

Mahawar and Devi [4]	76/M	NR	Maxillary sinus	+Cytokeratin	NR	No	EBRT, 7000 cGy	No	Currently under treatment

Rahim et al. [18]	32/F	Chinese	Nasopharynx	+MNF 116	NR	Endoscopic excision	NR	NR	NR
				+EBV					

Wöckel and Wernert [19]	56/M	NR	Meatus of the nose	+Keratin	T1N0M0 stage I	Excision	No	No	No recurrence
				−EBV					

Bonnerup et al. [10]	77/M	Caucasian	Sinonasal	+Pankeratin	T4aN2cMI stage IVC	NR	NR	Cisplatin and docetaxel	Excellent tumor response after two cycles
				+CK5/6					
				+EMA (patchy)					
				+EBV, strong and diffuse					

Trabelsi et al. [9]	58/M	Tunisian	Nasal septum with orbital invasion	+Cytokeratin, EMA	T3N0M0 stage III	No	72 Gy	Adriamycin and cisplatin	12-month follow-up, no recurrence
				+EBV latent membrane protein 1					

M: male; F: female; NR: not reported; IHC: immunohistochemistry; EPV: Epstein–Barr virus; CK: cytokeratin; EMA: epithelial membrane antigen; Gy: gray; CEA: carcinoembryonic antigen; LN: lymph node; IMRT: intensity-modulated radiotherapy; EBRT: external beam radiotherapy.

## Data Availability

The data used to support the findings of this study are included within the article.
